# Genome-wide identification and expression analysis of the *Hsp20*, *Hsp70* and *Hsp90* gene family in *Dendrobium officinale*

**DOI:** 10.3389/fpls.2022.979801

**Published:** 2022-08-10

**Authors:** Hongman Wang, Zuqi Dong, Jianbing Chen, Meng Wang, Yuting Ding, Qingyun Xue, Wei Liu, Zhitao Niu, Xiaoyu Ding

**Affiliations:** ^1^College of Life Sciences, Nanjing Normal University, Nanjing, China; ^2^Jiangsu Provincial Engineering Research Center for Technical Industrialization for Dendrobium, Nanjing, China; ^3^College of Forestry, Beijing Forestry University, Beijing, China; ^4^Shenzhen Key Laboratory for Orchid Conservation and Utilization, The National Orchid Conservation Center of China and The Orchid Conservation and Research Center of Shenzhen, Shenzhen, China

**Keywords:** *Dendrobium officinale*, gene family, heat shock proteins, genome-wide analysis, expression profiles

## Abstract

*Dendrobium officinale*, an important orchid plant with great horticultural and medicinal values, frequently suffers from abiotic or biotic stresses in the wild, which may influence its well-growth. Heat shock proteins (*Hsp*s) play essential roles in the abiotic stress response of plants. However, they have not been systematically investigated in *D. officinale*. Here, we identified 37 *Hsp20* genes (*DenHsp20*s), 43 *Hsp70* genes (*DenHsp70*s) and 4 *Hsp90* genes (*DenHsp90*s) in *D. officinale* genome. These genes were classified into 8, 4 and 2 subfamilies based on phylogenetic analysis and subcellular predication, respectively. Sequence analysis showed that the same subfamily members have relatively conserved gene structures and similar protein motifs. Moreover, we identified 33 pairs of paralogs containing 30 pairs of tandem duplicates and 3 pairs of segmental duplicates among these genes. There were 7 pairs in *DenHsp70*s under positive selection, which may have important functions in helping cells withstand extreme stress. Numerous gene promoter sequences contained stress and hormone response *cis*-elements, especially light and MeJA response elements. Under MeJA stress, *DenHsp20*s, *DenHsp70*s and *DenHsp90*s responded to varying degrees, among which *DenHsp20-5,6,7,16* extremely up-regulated, which may have a strong stress resistance. Therefore, these findings could provide useful information for evolutional and functional investigations of *Hsp20*, *Hsp70* and *Hsp90* genes in *D. officinale*.

## Introduction

Plants are commonly exposed to biotic and abiotic stresses, e.g., drought, heat, and various pathogens, which could cause adverse effects on their growth and development (Zhang et al., 2022). To overcome these difficulties, plants have evolved their own “stress tolerance system,” which attracted intense attention from botanists, leading to numerous functional genomic studies related to plant stress tolerance published, particularly in temperature stresses (Dangi et al., 2018). For example, Heat shock proteins (*Hsp*s) help newly synthesized proteins to fold, or to protect proteins that might misfold and thereby lose their potential functional conformation during a heat stress event. However, stresses in nature rarely come alone. Heat stress is commonly associated with high light and drought, but also promotes the spreading of pathogens and pests, leading to serious harm to plants (Jacob et al., 2017). Thus, *Hsp*s, which were proved to be involved in multiple stress resistance, especially in abiotic stresses, have an important effect on thermomorphogenesis (Lin et al., 2022). Heat shock proteins, originally only described in relation to heat shock (Ritossa, 1962), were actually induced by a wide variety of stresses, including exposure to cold, osmotic, drought, salt, UV, high light, wound healing, tissue remodeling, or biotic stresses (Lindquist and Craig, 1988; Vierling, 1991; Boston et al., 1996). Therefore, *Hsp*s play a great role in alleviating the injury caused by stresses.

According to previous studies, *Hsp*s can be grouped into six families including *Hsp100*s/ClpB, *Hsp90*s, *Hsp70*s/DnaK, *Hsp60*s, *Hsp40*s/DnaJ and *Hsp20*s based on their molecular weight and sequence homology (Wang et al., 2004; Waters, 2013). Among them, three gene families of *Hsp20*, *Hsp70* and *Hsp90* are the most important in plants. *Hsp20*, the most variable and diversified family in plants, was considered the most produced protein under heat stress conditions in many higher plants (Vierling, 2003; Charng et al., 2006; Basha et al., 2012). *Hsp70*, the most abundant heat shock proteins in eukaryotic cells, was expressed and accumulated under many stresses, such as drought stress (Cho and Choi, 2009), high-salinity stress (Wang et al., 2008), heavy metal stress (Tukaj et al., 2011) and virus stress (Aparicio et al., 2005), suggesting that *Hsp70* can increase the resistance of plants to various biotic or abiotic stresses. Unlike *Hsp20* and *Hsp70* genes, which mainly play a role in plant growth and development and response to environmental stress, *Hsp90*s play essential roles in plant immunity (Shirasu, 2009; Kadota and Shirasu, 2012). For example, Wang et al. (2011) have found that wheat plants over-expressing the *TaHsp90.2* and *TaHsp90.3* from *Hsp90* genes showed significant resistance to stripe rust. In rice, a chaperone complex consisting of cytosolic *Hsp90* and its co-chaperone *Hop/Sti1* participates in chitin responses and anti-fungal immunity (Chen et al., 2010).

In recent years, as more plant genomes have been assembled and reported, the *Hsp20*, *Hsp70* and *Hsp90* gene family were identified across many plant species, such as *Arabidopsis thaliana* (Krishna and Gloor, 2001; Lin et al., 2001; Scharf et al., 2001), *Oryza sativa* (Ouyang et al., 2009; Sarkar et al., 2012; Xu et al., 2012) and *Triticum aestivum* (Muthusamy et al., 2017; Lu et al., 2020; Lai et al., 2021). *Dendrobium officinale* Kimura et Migo, an endangered orchid endemic to China, has great horticultural and medicinal values (Niu et al., 2018; Zhu et al., 2018; Li et al., 2020). It grows in adverse conditions, e.g., epiphytic on cliffs or tree trunks, and distributed at high altitudes above 1,200 m. Moreover, *D. officinale* is susceptible to pests and diseases such as anthracnose, blackspot and phytophthora mealybug, which may have a negative influence on its well-growth. However, even in such harsh habitats, *D. officinale* still can grow well and accumulate important medicinal substances. Therefore, it is important to make clear the mechanism of its stress resistance, especially the response of *Hsp* genes to adversity. However, to date, the evolution and functions of *Hsp20*, *Hsp70* and *Hsp90* gene family in *D. officinale* still remains unclear. With the availability of chromosome-level genome sequence of *D. officinale* (Niu et al., 2020), it is now possible to conduct full study the three *Hsp* gene families in *D. officinale*.

Here, we used bioinformatics methods to identify *Hsp20*, *Hsp70* and *Hsp90* genes from *D. officinale* genome and uncover their sequence features, chromosomal positions, phylogenetic relationships, gene duplication events and syntenic analysis. Moreover, *cis*-elements, expression profiles, the protein three-dimensional (3D) structures and their protein–protein interaction (PPI) networks were all predicted to explore the possible biological functions of *Hsp* genes. The results would provide valuable information for further investigations of the *Hsp20*, *Hsp70* and *Hsp90* gene family in *D. officinale*.

## Materials and methods

### Identification of *Hsp20*, *Hsp70* and *Hsp90* genes in *Dendrobium officinale* genome

The Hidden Markov models (HMMs) profiles of *Hsp20* (PF00011), *Hsp40* (PF00226), *Hsp70* (PF00012) and *Hsp90* (PF00183), were downloaded from the protein family database (Pfam).^1^ This analysis was used for the search to recognizing candidate proteins with an E-value of 1e-5 by HMMER v3.2.1. Meantime, *Hsp20*, *Hsp40*, *Hsp60*, *Hsp70*, *Hsp90* and *Hsp100* protein sequences of *A. thaliana* and *O. sativa* from Scharf (Krishna and Gloor, 2001; Lin et al., 2001; Scharf et al., 2001), Ouyang (Ouyang et al., 2009; Sarkar et al., 2012; Xu et al., 2012) and (Ratheesh et al., 2012)^2^ were used as queries to search for *D. officinale* proteins using BLASTP. Ultimately, the protein sequences were integrated by both the above methods. Here, we chose *Hsp20*, *Hsp70* and *Hsp90* gene family in *D. officinale* for further analysis. So, the output putative *DenHsp20*s, *DenHsp70*s and *DenHsp90*s were submitted to Pfam (see Footnote 1), NCBI-CDD^3^ and SMART^4^ to confirm conserved domains. In addition, the protein molecular weight (kDa), aliphatic index (AI), theoretical isoelectric point (pI) and grand average of hydropathicity (GRAVY) were estimated with ExPASy software (Wilkins et al., 1999). The chromosome locations were analyzed and displayed by TBtools v1.6 (Chen et al., 2020).

### Phylogenetic relationship, gene structure and motifs and domains

Multiple sequence alignments of *Hsp20*, *Hsp70* and *Hsp90* full-length amino acid sequences derived from *D. officinale*, *A. thaliana* and *O. sativa* were performed with MAFFT v7.487 software (Katoh and Dtandley, 2013). And the maximum likelihood phylogenetic trees were constructed by RAxML v1.3 with a bootstrap value of 1,000. The conserved motifs were determined by MEME,^5^ with default parameters, except that the number of motifs of 20 was specified. Additionally, the exon-intron structure of each sequence was displayed by GSDS software online.^6^ The different characteristic domains of the three gene families were aligned with previous studies (Krishna and Gloor, 2001; Lin et al., 2001; Scharf et al., 2001) and visualized by DNAMAN_9 software.

### Gene duplication, syntenic analysis and non-synonymous and synonymous calculation

Firstly, the genomic DNA sequences of the *Hsp20*, *Hsp70* and *Hsp90* genes of *D. officinale* were aligned using BLASTN with an E-value of 1e-20. Then the gene duplications were identified with MCScanX using BLASTN results. The duplication events of these genes were visualized with Circos (Krzywinski et al., 2009). Besides, the syntenic blocks between *D. officinale* and other plant genomes were detected and displayed with MCScanX (cscore≥0.7). KaKs_Calculator 2.0 (Wang et al., 2010) was used to estimate Non-synonymous (Ka), synonymous (Ks) and Ka/Ks ratios. Commonly, Ka/Ks > 1 indicates positive selection, Ka/Ks < 1 indicates negative selection, and Ka/Ks = 1 indicates neutral selection (Wang et al., 2010).

### Promoter analysis

The upstream 1,500 bp genomic DNA sequences of *Hsp20*, *Hsp70* and *Hsp90* genes were extracted as putative promoters. Then they were submitted to online PlantCare database^7^ to analyze the putative *cis*-elements. FIMO (Noble, 2011) in MEME software toolkit was used to predict heat shock responsive elements (HSEs) using sequence module nTTCnnGAAnnTTCn or nGAAnnTTCnnGAAn (Sarkar et al., 2009; Lopes-Caitar et al., 2013). Total *cis*-elements in promoter sequences were visualized by TBtools v1.6 software (Chen et al., 2020).

### Expression profiles

To analyze the expression patterns of these identified genes in *D. officinale*, we searched the NCBI SRA database^8^ for RNA-sequence data from four different tissues (root, stem, leaf, and flower) with the accession IDs SRR2014227, SRR2014230, SRR2014236, SRR2014246, SRR2014297, SRR2014325, SRR2014396, and SRR2014476 (Chen et al., 2017). Firstly, the download RNA-sequence data were converted to fastq format *via* fastq-dump of SRA toolkit.3.0.0. Then the clean reads were aligned to the *D. officinale* genome, and mapping by Hisat2 v2.2.1. And the data were sam to bam by SAMtools v1.14. The FPKM value of *DenHsp20*s, *DenHsp70*s and *DenHsp90*s were calculated by StringTie v2.2.0 to estimate the transcript abundances. The heat map was constructed by the pheatmap package in RStudio v1.4.1717 to visualize the expression.

### Quantitative real-time PCR analysis

Total RNA was extracted by an EASY spin Plant RNA Kit (Aidlab, China). First–strand cDNAs were synthesized using HiScript® III–RT SuperMix for qPCR (Vazyme, China). Primers (Supplementary Table S6) were designed using Snapgene software. qRT-PCR was performed using ABI-7500 Connect Real-Time PCR Detection System. cDNAs were diluted to 200 ng with 1 μl template in a reaction volume of 20 μl, run in three technical replicates. PCR amplification programs were used as follows: 95°C for 30 s followed by 40 cycles of 95°C for 10 s, 60°C for 30 s, and 60°C for 15 s. The expression data were calculated by the 2^-∆∆CT^ method (Livak and Schmittgen, 2002).

### Three-dimensional protein structure prediction and protein–protein interaction network

The tertiary structures of *Hsp20*, *Hsp70* and *Hsp90* proteins in *D. officinale* were predicted with SWISS-MODEL.^9^ The *Hsp20*, *Hsp70* and *Hsp90* protein sequences were aligned to STRING database^10^ online to predict the relationships. Cytoscape v3.7.2 software (Shannon et al., 2003) was used to visualize the regulatory networks.

## Results

### Identification and distribution of *Hsp20*, *Hsp70* and *Hsp90* genes in *Dendrobium officinale*

A total of 165 *Hsp* genes, with 37 *Hsp20* genes, 70 *Hsp40* genes, 7 *Hsp60* genes, 43 *Hsp70* genes, 4 *Hsp90* genes and 4 *Hsp100* genes, were identified from *D. officinale* genome sequence using HMMER and BLASTP method. Here, we chose *Hsp20*, *Hsp70* and *Hsp90* gene family in *D. officinale* for further analysis (Table 1; Supplementary Table S1). The characters among the three kinds of *Hsp* genes were variable. For example, Hsp70 proteins contains more variable amino acid numbers (from 45 of DenHsp70-21 to 657 of DenHsp70-8) than *Hsp*20 (from 73 of DenHsp20-14 to 294 of DenHsp20-10) and Hsp90 (from 428 of DenHsp90-4 to 510 of DenHsp90-3). The protein molecular weight (MW) of Hsp70 ranged from 5.1 kDa (DenHsp70-14) to 73.4 kDa (DenHsp70-8), higher than Hsp20 (from 8.4 kDa of DenHsp20-14 to 33.3 kDa of DenHsp20-10) and Hsp90 (from 49.4 kDa of DenHsp90-4 to 59.3 kDa of DenHsp90-3). In contrast, the isoelectric point (PI) of Hsp90 (from 78.98 of DenHsp90-2 to 84.95 of DenHsp90-4) was higher than that of Hsp70 (from 4.4 of DenHsp70-9 to 8.99 of DenHsp70-25) and Hsp20 (from 4.92 of DenHsp20-12 to 9.34 of DenHsp20-11; Table 1; Supplementary Table S1).

**Table 1:** The characteristics of *Hsp20*, *Hsp70* and *Hsp90* members identified in *Dendrobium officinale.*

Gene Family	Protein	Molecular weight(kDa)	Theoretical pI	Aliphatic index	Grand average of hydropathicity(GRAVY)
*Hsp20*	73 ~ 294	8.4 ~ 33.3	4.92 ~ 9.34	81.36 ~ 114.94	−0.558 ~ 0.452
*Hsp70*	45 ~ 657	5.1 ~ 73.4	4.4 ~ 8.99	54.63 ~ 99.54	−1.023 ~ −0.119
*Hsp90*	428 ~ 510	49.4 ~ 59.3	78.98 ~ 84.95	78.98 ~ 84.95	−0.786 ~ −0.591

**Figure 1:**
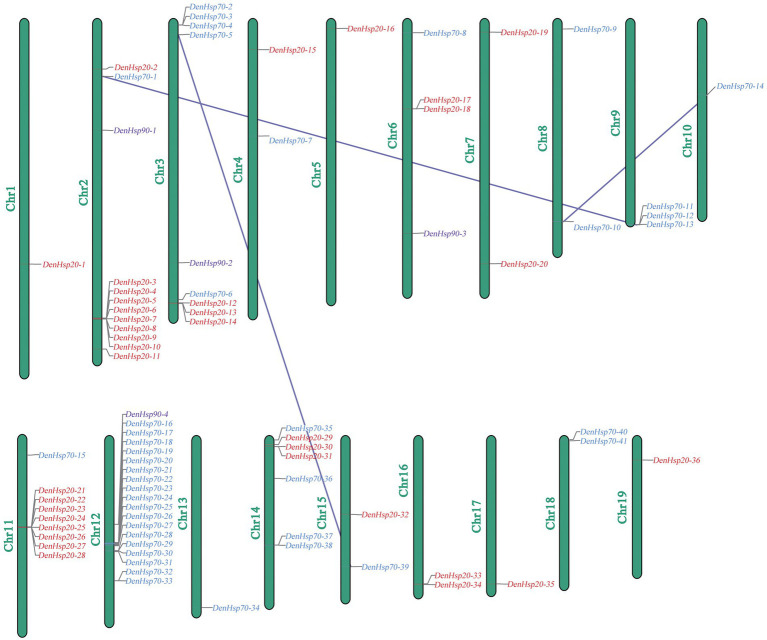
Chromosomal distributions of the identified *Hsp20*, *Hsp70* and *Hsp90* genes in *Dendrobium officinale*. Red, blue, purple colors represent *Hsp20*, *Hsp70* and *Hsp90* genes, respectively. Three lines represent three tandem duplicate pairs.

Among the 19 assembled chromosomes of *D. officinale*, *DenHsp20*s, *DenHsp70*s and *DenHsp90*s were distributed on 13 chromosomes, 13 chromosomes and 4 chromosomes, respectively. As shown in Figure 1, most *Hsp20* genes were located on Chr2 and Chr11. Chr12 contained the most *Hsp70* genes, although it was not the longest chromosome. Gene clusters could be observed on Chr2, Chr11 and Chr12. Remarkably, most *Hsp* genes belonging to the same subfamilies were mapped on the same chromosome. This shows that tandem duplication events played an important role during the expansion of the *Hsp* family in *D. officinale*.

### Phylogenetic analysis of the *Hsp20*s, *Hsp70*s and *Hsp90*s

**Figure 2:**
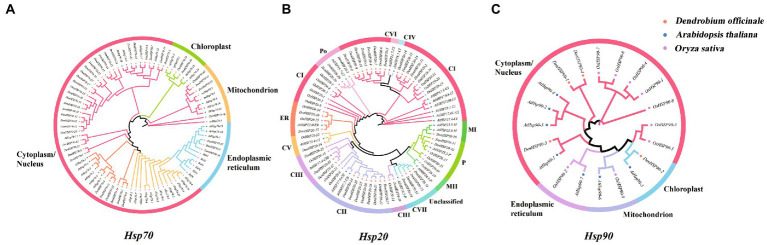
**(A–C)** represent the phylogenetic relationships of *Hsp20*, *Hsp70* and *Hsp90* genes in *D. officinale*, *A. thaliana* and *O. sativa*, respectively. Three maximum likelihood phylogenetic trees were constructed by RAxML with 1000 bootstraps. Orange, green, purple colors represent *Hsp20*, *Hsp70* and *Hsp90* protein sequences from *D. officinale* (Den), *A. thaliana* (At) and *O. sativa* (Os), respectively. Different subfamilies are shaded with different colors.

To explore the evolutionary relations of *Hsp* genes of *D. officinale*, phylogenetic trees of *Hsp20*, *Hsp70* and *Hsp90* genes were constructed, respectively, with *Hsp* genes from *A. thaliana* and *O. sativa* as outgroups (Figure 2).

The 37 *DenHsp20*s were classified into 8 distinct subfamilies, including 13 CIs, 8 CIIs, 4 CIIIs, 1 CV, 2 ERs, 1 MI, 2 Ps, 2 Pos, and 4 unclassified, which were consistent with the results of previous studies where CI was the largest subfamily (Figure 2A). In addition to being consistent with the theory that the CIV subfamily exists only in dicotyledons as confirmed by previous studies, the CVI and CVII subfamilies of out phylogenetic tree also exist only in the dicotyledonous plant *A. thaliana*. The 43 *DenHsp70*s were classified into four subfamilies according to Wolf and CELLO subcellular localization prediction, 35 *DenHsp70*s in Cytoplasm/ Nucleus, 2 in Chloroplast, 3 in Mitochondrion, and 3 in Endoplasmic reticulum (Figure 2B). The *DenHsp90*s were divided into the same four subfamilies by the same method as *DenHsp70*s, and the four *DenHsp90*s were present in only two of these subfamilies, three in the Cytoplasm/ Nucleus and one in the Chloroplast (Figure 2C). By comparison, *AtHsp90*s was present in all four subfamilies, while *OsHsp9*0s was present in three subfamilies except Chloroplast. Overall, the cytoplasm has the largest number of *Hsp* genes and is probably the main working region for heat shock proteins.

### Gene structure and motif analysis of *DenHsp20*s, *DenHsp70*s and *DenHsp90*s

**Figure 3:**
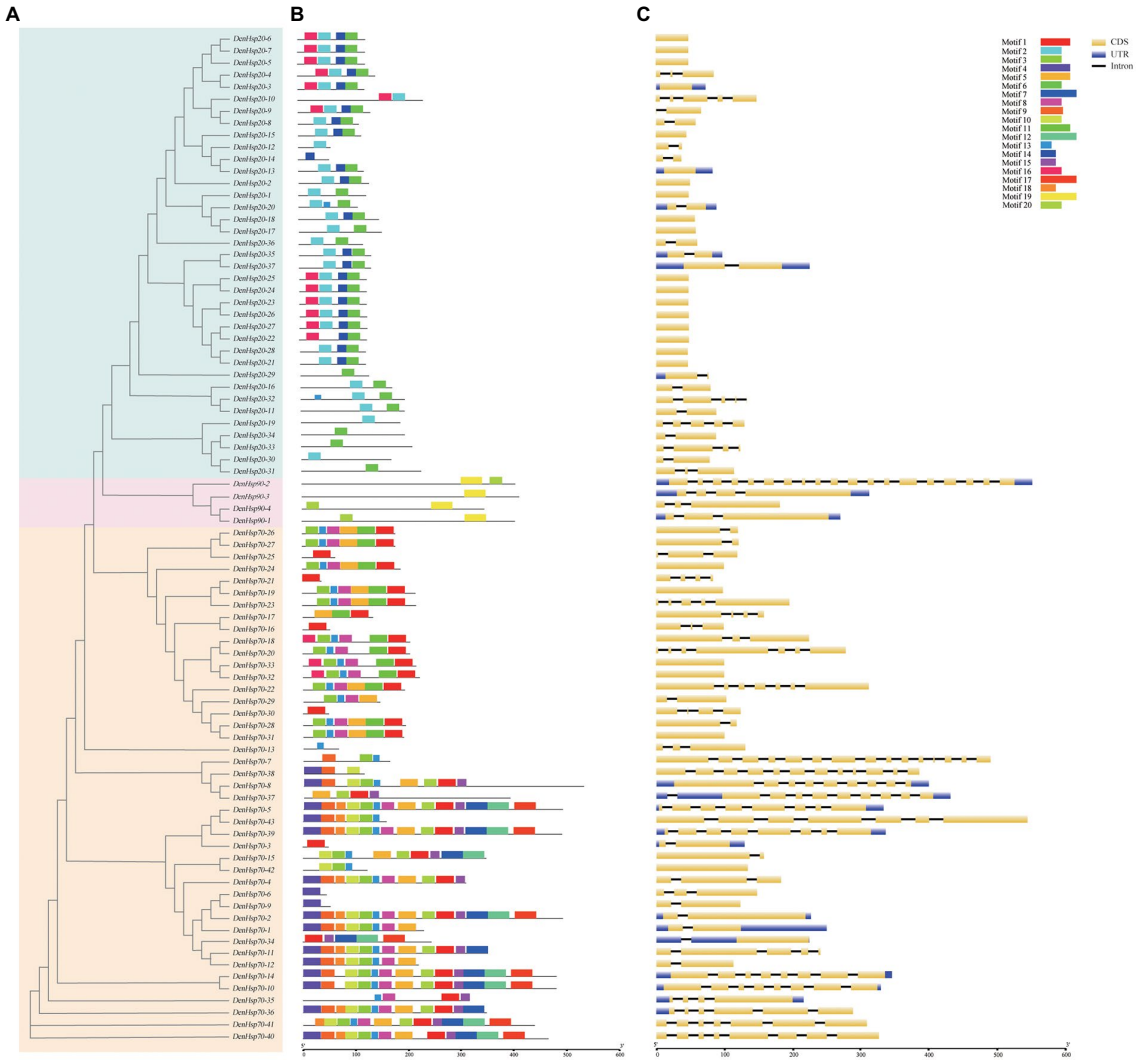
Phylogenetic relationships **(A)**, conserved motifs **(B)** and exon-intron structures **(C)** of *Hsp20*, *Hsp70* and *Hsp90* genes in *Dendrobium officinale*. **(A)** Blue, red, orange colors represent *Hsp20*, *Hsp90* and *Hsp70* genes. **(B)** The conserved motifs of 84 proteins were identified using MEME and visualized by TBtools. Different colors represent 20 different motifs. **(C)** Yellow and blue boxes are, respectively, indicating CDS and UTR. And the black lines indicate introns.

A phylogenetic tree was constructed from 84 amino acid sequences of *DenHsp20*s, *DenHsp70*s and *DenHsp90*s (Figure 3A). To resolve the motif composition of *DenHsp20*s, *DenHsp70*s and *DenHsp90*s, the 84 sequences were submitted to the MEME website. A total of 20 motifs were predicted with length ranging from 15 to 50 amino acids (Figure 3B). According to the detailed motif sequence, we found that the *DenHsp20*s, *DenHsp70*s and *DenHsp90*s had different motifs (As shown in Supplementary Figure 1). (i) Among the 20 motifs, Motif 2 and Motif 6 were widespread on almost all the *DenHsp20*s. Motif 14 and Motif 16 were specific to subfamilies CI and CII. (ii) The *DenHsp70*s had the most conserved motifs. Motif 3, Motif 5, Motif 8 and Motif 13 were widely widespread on the *DenHsp70*s.(iii) Motif 19 was widespread on all the *DenHsp90*s. (iiii) Especially, Motif 13 was on the *DenHsp20-20* and *DenHsp20-32* in addition to *DenHsp70*s.

Exon-intron structure provides an important clue for gene’s functional diversification. We investigated the exon-intron pattern of the 37 *DenHsp20*s, 43 *DenHsp70*s and 4 *DenHsp90*s according to *D. officinale* genome annotation information (Figure 3C). The results showed that the *DenHsp20*s were intron-less. It was obvious that genes in the same subfamily showed similar gene structures. Most members in subfamily CII of *DenHsp20*s had no introns. These results indicated that the structures of *DenHsp20*s were more conserved than those of *DenHsp70*s and *DenHsp90*s.

The basic structure of *Hsp*s is conserved throughout the eukaryotic kingdom. Domain analysis helps to better understand different *Hsp* genes (Supplementary Figure 2). The *Hsp20* sequences contain the central conserved domain, the α-crystallin domain (ACD). *Hsp70* is composed of two domains: the ATPase domain, also referred as nucleotide binding domain, NBD (Flacherty et al., 1990), and the substrate binding domain (SBD; Zhu et al., 1996). Eukaryotic *Hsp90* proteins contain 2 highly conserved domains: the adenosine triphosphate (ATP)-binding domain at the N-terminus and the highly charged (glutamic acid-rich) linker region (Xu et al., 2012).

### Gene duplication and syntenic analysis of *DenHsp20*s, *DenHsp70*s and *DenHsp90*s

**Figure 4:**
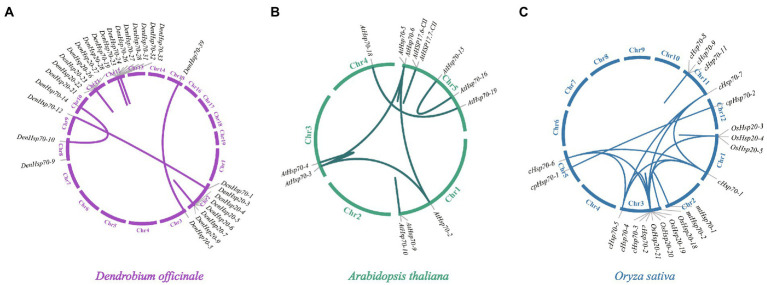
Schematic representations of the gene duplications of *Hsp20*, *Hsp70* and *Hsp90* genes from three different plants. **(A–C)** represent the syntenic gene pairs in *D. officinale*, *A. thaliana* and *O. sativa*, respectively.

Synteny analysis was conducted to the *DenHsp20*s, *DenHsp70*s and *DenHsp90*s using BLASTN and MCScanX to investigate gene duplication events. A total of 20 and 13 pairs of paralogous genes were detected among *DenHsp20*s and *DenHsp70*s, respectively (Figure 4A). Among them, there were 3 pairs of segmental duplications from *DenHsp70*s located on the *D. officinale* chromosomes (i.e., Chr2, Chr8, Chr10 and Chr15). While the remaining 30 pairs of tandem duplicates, with 20 pairs from *DenHsp20*s and 10 pairs from *DenHsp70*s, were focused on Chr2, Chr11 and Chr12 (Supplementary Table S3).

Ka/Ks values of *DenHsp20*s’ and *DenHsp70*s’ duplicate gene pairs were calculated to evaluate the driving force underlying the *HSP* gene’s evolution. The results showed that Ka/Ks values of *DenHsp20* duplicate genes ranged from 0.001 ~ 0.624, indicating that *Hsp20* family was under negative selection during the evolution process (Supplementary Table S2). Among *DenHsp70*s paralogs, 7 pairs, *DenHsp70-24* and *DenHsp70-26*, *DenHsp70-26* and *DenHsp70-27*, *DenHsp70-28* and *DenHsp70-32*, *DenHsp70-28* and *DenHsp70-33*, *DenHsp70-31* and *DenHsp70-32*, *DenHsp70-31* and *DenHsp70-33*, *DenHsp70-32* and *DenHsp70-33*, were positively selected, and the remaining pairs of genes experienced a negative selection (Table 2).

**Table 2:** Ka, Ks and Ka/Ks values for duplication gene pairs in *Dendrobium officinale.*

Seq_1	Seq_2	Ka	Ks	Ka/Ks	Duplication type
*DenHsp70-24*	*DenHsp70-26*	0.25634	0.233194	1.09926	Tandem duplication
*DenHsp70-26*	*DenHsp70-27*	0.0434161	0.0430908	1.00755	Tandem duplication
*DenHsp70-28*	*DenHsp70-32*	0.201249	0.122347	1.6449	Tandem duplication
*DenHsp70-28*	*DenHsp70-33*	0.209309	0.120741	1.73354	Tandem duplication
*DenHsp70-31*	*DenHsp70-32*	0.1781	0.0776771	2.29282	Tandem duplication
*DenHsp70-31*	*DenHsp70-33*	0.184554	0.0753664	2.44875	Tandem duplication
*DenHsp70-32*	*DenHsp70-33*	0.065504	0.02501	2.61911	Tandem duplication

Synonymous (Ks) and non-synonymous (Ka) substitution rates of duplicate gene pairs (Ka/Ks ratios).

To further understand the replication event of the *DenHsp20*s and *DenHsp70*s, the replication events were compared between *D. officinale* and two other species (*A. thaliana* and *O. sativa*). The analysis also demonstrated that segmental duplication *Hsp20* and *Hsp70* gene pairs were found in genomes of *A. thaliana* (6 pairs) and *O. sativa* (12 pairs; Figures 4B,C).

**Figure 5:**
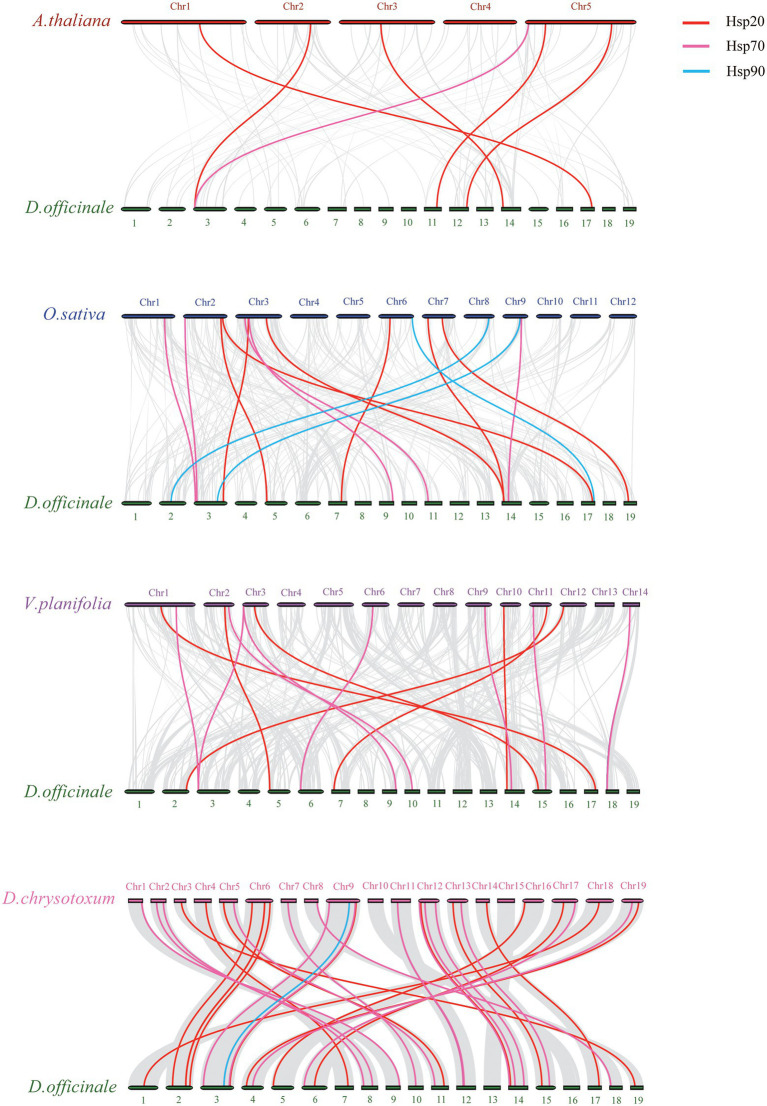
Collinearity analysis of *Hsp20*, *Hsp70* and *Hsp90* genes between *Dendrobium officinale* and four other plants, including *A. thaliana*, *O. sativa*, *V. planifolia* and *D. chrysotoxum*. Gray lines indicate the collinear blocks. Syntenic genes of *Hsp20*, *Hsp70* and *Hsp90* gene family are exhibited with red, pink and blue lines, respectively.

Moreover, we analyzed the collinearity of *Hsp20*, *Hsp70* and *Hsp90* genes between *D. officinale* and four other plants (Figure 5). Collinearity analysis showed that the homologous genes between *D. officinale* and *D. chrysotoxum* were the most abundant, with 37 homologous gene pairs, followed by *Vanilla planifolia* (15 homologous gene pairs), *O. sativa* (15 homologous gene pairs) and *A. thaliana* (6 homologous gene pairs). *DenHsp20-35* existed syntenic genes across the four species, and it is speculated that the gene may have originated from a common ancestor before the divergence of monocotyledons and dicotyledons with conserved and important functions. Excluding the dicotyledonous plant *A. thaliana*, *DenHsp70-36*, existed across the other three monocotyledons, may have been relatively conserved during monocotyledon evolution. In the collinearity analysis between *D. officinale* and *D. chrysotoxum*, the gene *KAH0459475* of *D. chrysotoxum* had homologous pairs with three *HSP70* genes (*DenHsp70-17*, *DenHsp70-28*, *DenHsp70-31*) in *D. officinale*, indicating that there was a duplication of the *DenHsp70*s.

### Analysis of *DenHsp20*s, *DenHsp70*s and *DenHsp90*s promoter

**Figure 6:**
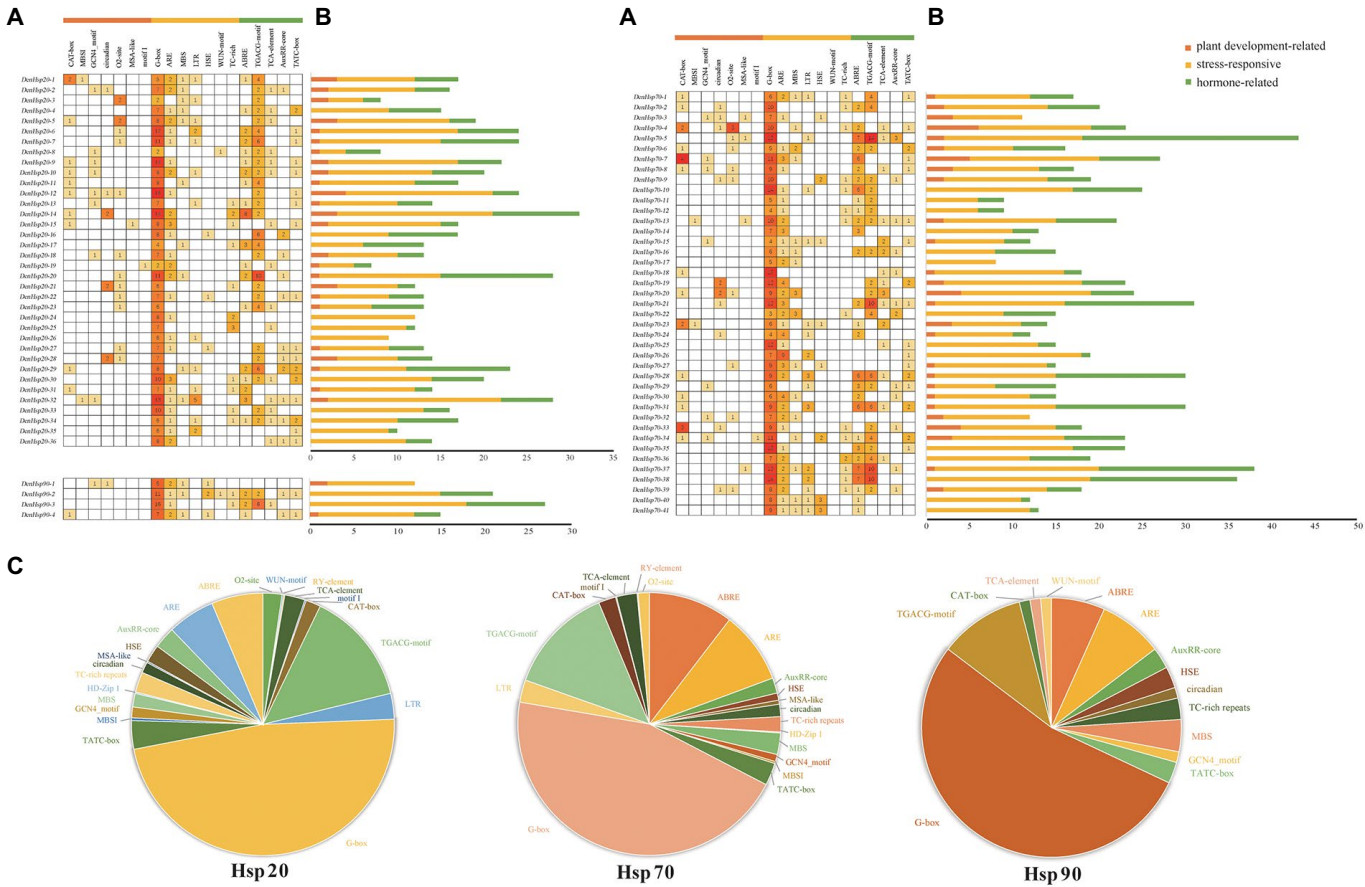
Information of *cis*-acting elements in *Hsp20*, *Hsp70* and *Hsp90* genes of *Dendrobium officinale*. **(A)** The gradient orange colors and numbers in the grid indicate the number of different *cis*-elements. **(B)** The different colors histogram indicates the number of *cis*-elements in each category. **(C)** The ratio of different *cis*-acting elements in *Hsp20*, *Hsp70* and *Hsp90* genes is shown as pie charts.

To better understand the potential function of *DenHso20*s, *DenHsp70*s and *DenHsp90*s, the *cis*-acting elements in the promoter regions were identified and analyzed. After removing non-functional terms, a total of 1,477 *cis*-acting elements in the promoter regions of *DenHsp20*s, *DenHsp70*s and *DenHsp90*s were classified into three categories of *cis*-elements, which are linked to plant growth and development (meristem expression, zein metabolism regulation, circadian control, etc.), stress responsiveness (light, anaerobic, low-temperature, drought, heat stress wound, and defense and stress), and phytohormone responsiveness (MeJA, abscisic acid, auxin, gibberellin and salicylic acid; Figure 6; Supplementary Figure 2; Supplementary Table S3).

In *DenHsp20*s, most of the *cis*-elements were related to the stress responsiveness category (389/608), followed by the phytohormone responsiveness category (173/608) and plant growth and development category (46/608). The proportion of three categories in *DenHsp70*s and *DenHsp90*s were similar to those in *DenHsp20*s. Notably, 20 HSEs (accounting for 1.42%) were distributed across 3 *DenHsp20*s, 7 *DenHsp70*s and 3 *DenHsp90*s, most of which were located in *DenHsp70*s. As shown in Supplementary Figure 2, light responsive elements were the most abundant, accounting for 46.55% of all elements, which were related to the special photosynthetic pathway of *D. officinale*. MeJA responsive elements were the second abundant, accounting for 13.27% of all the *cis*-elements. In addition, there were a large number of stress responsive elements in drought, low-temperature and other stress environment. These results suggested that the ubiquitous *cis*-elements could be involved in *DenHsp20*s, *DenHsp70*s and *DenHsp90*s expression regulation in response to multiple abiotic stresses.

### Expression analysis of *DenHsp20*s, *DenHsp70*s and *DenHsp90*s in different tissues and MeJA treat

**Figure 7:**
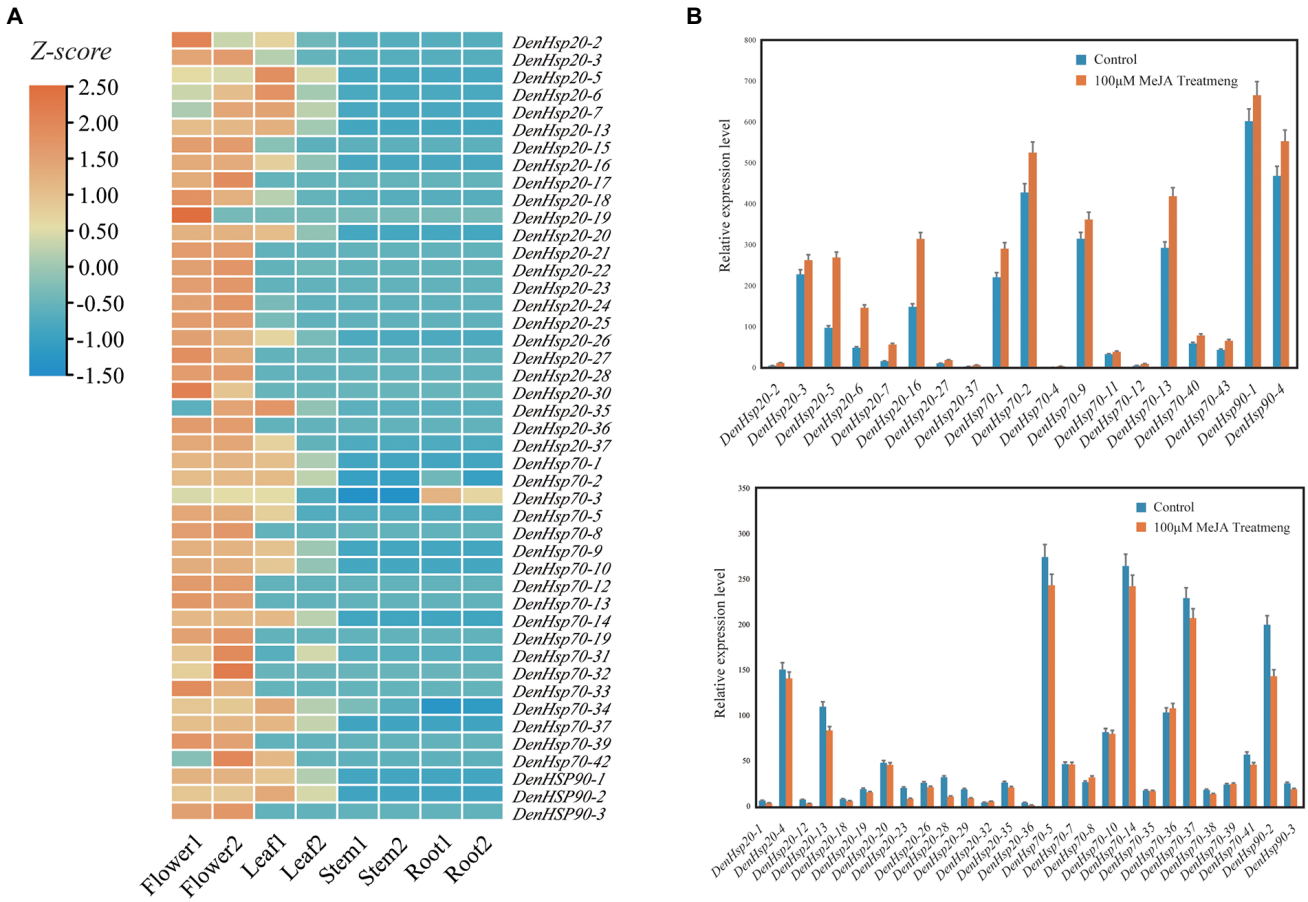
Expression analysis of *DenHsp20*s, *DenHsp70*s and *DenHsp90*s in different tissues and MeJA treat. **(A)** Expression profiles of *Hsp20*, *Hsp70* and *Hsp90* genes of *Dendrobium officinale* in different tissues including root, stem, leaf and flower. Z-score transformed FPKM values. **(B)** Relative expression levels of *DenHsp20*s, *DenHsp70*s and *DenHsp90*s under MeJA treatments.

The tissue-specific expression of *DenHsp20*s, *DenHsp70*s and *DenHsp90*s were analyzed to further studying gene functions. (Figure 7A). Among the 84 *Hsp* genes of *D. officinale*, 45 genes were expressed, including 24 *DenHsp20*s, 18 *DenHsp70*s and 3 *DenHsp90*s. While these genes were expressed in different tissue. The results showed a diversified tissue-specific expression, e.g., most genes were highly expressed in flowers and leaves, lowly expressed in roots and stems of *D. officinale*. *DenHsp70*-*36* was ubiquitously and highly expressed in every tissue, which was speculated that it may play a significant role in plant growth and development.

Analysis of *cis*-elements indicated that most of *Hsp* genes in *D. officinale* contained MeJA response elements. To investigate the potential role of *DenHsp20*s, *DenHsp70*s and *DenHsp90*s under MeJA treatment, we determined the expression pattern by qRT-PCR (Figure 7B). Among them, 19 genes were significantly up-regulated, 24 were downregulated, and the remaining genes had no significant change. Moreover, in these 19 up-regulated genes, the relative expression levels of 4 genes (*DenHsp20-5*, *DenHsp20-6*, *DenHsp20-7* and *DenHsp70-4*) were extremely up-regulated (more than 3-fold) under MeJA treatment, which may play a more important role in plant resistance to abiotic stresses.

### Three-dimensional protein structure of Hsp20, Hsp70 and Hsp90 proteins in *Dendrobium officinale*

Three-dimensional (3D) protein structures of DenHsp20 proteins, DenHsp70 proteins and DenHsp90 proteins were predicted with SWISS-MODEL. Subsequently, 62 successful models were defined by at least 30% identity of the target to a template, including 26 Hsp20 proteins, 16 Hsp70 proteins and 4 Hsp90 proteins (Supplementary Table S4). Compared with Hsp20 proteins (QMEAN DisCo Global from 0.60 to 0.78, GMQE from 0.42 to 0.78) and Hsp90 proteins (QMEAN DisCo Global from 0.74 to 0.78, GMQE from 0.75 to 0.82), the 3D structure models of Hsp70 proteins (QMEAN DisCo Global from 0.58 to 0.86, GMQE from 0.55 to 0.91) were of higher quality (Supplementary Table S4). A total of 46 different 3D structures, 26 for Hsp20 proteins, 4 for Hsp90 and 16 for Hsp70, were detected among *Hsp*s, which indicated the diversified biological functions for *Hsp* genes in *D. officinale*.

### Protein–protein interaction network of Hsp20, Hsp70 and Hsp90 proteins in *Dendrobium officinale*

**Figure 8:**
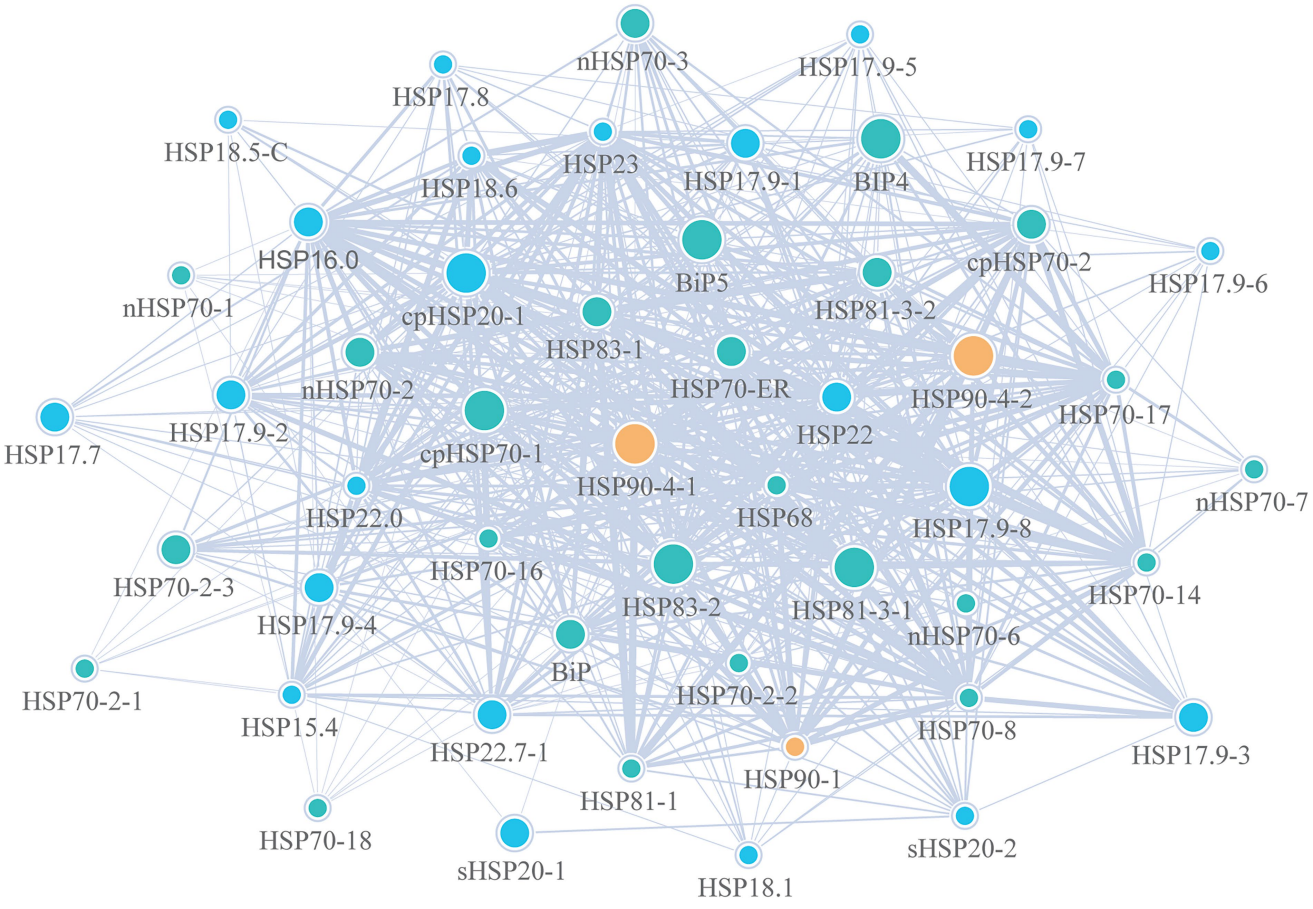
Protein–protein interaction (PPI) networks of Hsp20, Hsp70 and Hsp90 proteins in *Dendrobium officinale*. Blue, green and orange circles represent Hsp20, Hsp70 and Hsp90 proteins, respectively. The gradient circle size indicates the different degrees of importance.

To better understand the biological functions, the PPI networks were further analyzed to detect interactions among Hsp20 proteins, Hsp70 proteins and Hsp90 proteins and related proteins with the STRING website (Figure 8). Totally, 49 proteins and 661 connections were identified. Among the 661 connections, the Hsp90 proteins had the closest interaction with others, especially for the proteins of Hsp83. As shown in Supplementary Table S5, most of the proteins that interacted with Hsp proteins were the mediator of RNA polymerase II transcription subunits, such as MED14 and MED17. In addition, we also found that Hsp proteins may interact with stress-related transcription factors such as CYP and plant growth proteins like TCP. These results indicated that Hsp proteins of *D. officinale* have severe vital roles in multiple functions.

## Discussion

### Genome size, gene duplication and different evolution patterns may responsible for the quantity variance of *Hsp*s

The *Hsp* family, which plays an important role in the mechanism of plants responding to stresses and resisting the damage, is essential for plant species (Jiang et al., 2021). Though the sequences of *Hsp* genes were relatively conserved, the gene numbers of *Hsp* family varied greatly among plant lineages. For example, 17 genes of *Hsp*20 family were detected in *A. thaliana* (Scharf et al., 2001), while 39 and 117 genes were detected in *O. sativa* (Ouyang et al., 2009) and *T. aestivum* (Wang et al., 2017), respectively. In this study, a total of 84 *Hsp* genes, with 37 *DenHsp20*s, 43 *DenHsp70*s and 4 *DenHsp90*s, were identified from the chromosome-level genome sequences of *D. officinale*. We believed that three reasons may be responsible for the differences of different species and different *Hsp* families. Firstly, the varied gene number of *Hsp* family is correlated with the genome size variation. For example, *A. thaliana*, with a small genome size (125 Mb; Scharf et al., 2001) contained the lowest gene number (17 *Hsp20* genes) of *Hsp20* gene family, while *T. Aestivum* with large genome size (6.3 Gb; Wang et al., 2017), achieved the largest gene number (117 *Hsp20* genes) of *Hsp20* gene family. Secondly, gene duplication, which could multiply increase the quantity of genes, may be responsible to the gene number variation of *Hsp* gene family. In this study, a total of 30 gene duplications were investigated from the genome sequence, which increased the number of genes in the *Hsp* family by 25%. Moreover, genome expansion, especially for the expansion raised by WGD events may also cause the gene variation of the *Hsp* family. For example, the two rounds of WGD events that occurred in *D. officinale* have resulted in 3 genes increasing, which led to the number increase of the *Hsp* gene family. Compared with segmental duplications and tandem duplications accounted for a larger proportion in *D. officinale*, WGD was likely to enhance plant resistance by massively increasing the number of *Hsp70* genes. Thus, we speculated that this may improve its adaption to environmental stresses. Thirdly, the different evolution patterns in different plant lineage may also resulted in the quantity variance. For example, in monocotyledonous plant lineage, *Hsp20* genes undergone separate evolution after the divergence of monocots and dicots. As concluded from previous studies, the *Hsp20* genes of *A. thaliana* can be divided into 12 subfamilies (CI, CII, CIII, CIV, CV, CVI, CVII, MI, MII, P, Po, ER; Scharf et al., 2001; Ma et al., 2006; Siddique et al., 2008). In this study, there were no CIV, CVI, CVII and MII subfamilies detected in *D. officinale*. The CIV subfamily was only present in dicotyledons in previous studies, which may lead to changes in the number of *Hsp* genes (Siddique et al., 2008; Zhao et al., 2018; Yao et al., 2020). Moreover, this could be a proof for the different evolutionary relationships in monocotyledons and dicots (Cui et al., 2021). We speculated that monocotyledons may require more *Hsp20* genes to cope with environmental pressures. Therefore, genome size, gene duplication and different evolution patterns may be responsible for the quantity variance of *Hsp*s.

### Protein structures, distribution and tissue-specific expression patterns and interactions of *Hsp*s resulted in the diversity of biological functions

Previous studies have shown that heat shock proteins play a critical role in the molecular mechanisms such as plant development and defense against abiotic (Jiang et al., 2021). However, different *Hsp* gene families have different functions. Even the same gene family has different functions. For instance, *Hsp90* has been reported as a key regulator of normal growth and development in *Nicotiana benthamiana* and *A. thaliana* (Queitsch et al., 2002; Liu et al., 2004; Sangster and Queitsch, 2005; Sangster et al., 2007). While Zhang et al. (2013) analyzed the *Hsp90* genes of *Populus trichocarpa* and found that *Hsp*90 proteins expression was observed in most organisms in response to stress. Inferred from our comparative analysis, we believed that protein structures, distribution and tissue-specific expression patterns and interactions of *Hsp*s resulted in the diversity of biological functions.

Protein conformation is closely related to their biological functions. The diversity of protein structures may indicate the diversity of biological functions. In this study, a total of 46 different 3D structures among *Hsp* genes were detected, which indicated the diversified biological functions of *Hsp* genes. Moreover, the diversified distribution of *Hsp* genes, widely in the cytoplasm, nucleus and different organelles, may also be responsible for their diversified gene function. For example, previous studies have shown that (i) *Hsp*s are mainly located in the cytoplasm and respond to abiotic and biotic stresses (Park and Seo, 2015). (ii) *Hsp90C* functions in a chaperone complex of the chloroplast matrix, facilitating membrane transport during protein entry into organelles (Inoue et al., 2013). (iii) Mitochondrial heat shock protein 70 (mt*Hsp*70) functions in the unfolding, translocation, and folding of imported proteins (Voisine et al., 1999). In this study, in *D. officinale*, according to the result of subcellular localization, *DenHsp20*s were located in the cytosol, ER, mitochondria and chloroplast. While, *DenHsp70*s and *DenHsp90*s were located in Cytosol, ER, nucleus, mitochondria and chloroplast. These results implied that *Hsp* genes had various functions in *D. officinale*. For instance, we found that *Hsp* genes present tissue-specific expression patterns in *D. officinale*. Most *Hsp* genes were highly expressed in flowers and leaves, while they were rarely expressed in roots and stems.

Additionally, there are interactions between different proteins. For example, *Hsp*90 physically interacts with many cochaperones, including different *Hsp* families, to recruit and interact with diverse substrate proteins, leading to alteration of cellular processes. As expected, in this study, the integrated PPI network found that the majority of the Hsp20, Hsp70 and Hsp90 proteins were enriched. Among them, Hsp proteins were closely related to each other and might share biological functions. In addition to the Hsp proteins, some MED proteins and stress-related proteins were also enriched in the networks, which resulted in the diversity of biological functions.

Therefore, these results indicated that *Hsp*s had various functions in *D. officinale*. Moreover, these also provided ideas for further research on the biological functions of *Hsp* genes.

### Adverse habitat, special photosynthetic pathway and heat dissipation possibility influence the evolution of *DenHsp70*s

*Hsp* genes were highly conserved in their gene sequences, especially for *Hsp70* (Sharma and Masison, 2009). Thus, we evaluated that the evolution rate of protein coding genes of *Hsp* genes to see if they were undergone adaptive selection. Indeed, there were 6 gene pairs of *DenHsp70*s under positive selection (Ka/Ks > 1). We speculated that three reasons may possibly influence the evolution of *DenHsp70*s.

Firstly, previous studies showed that *Hsp70* was the main and highly conserved protein activated by stress in living organisms, which could help the cells to withstand extreme stress (Usman et al., 2017). *D. officinale* grows in an adverse habitat, such as epiphytic on cliffs or tree trunks, and distributed at high altitude above 1,200 m (Yu et al., 2020). In extreme environments, plants often need *Hsp70*s to respond to stress quickly, so *Hsp70*s is often subjected to stronger selection pressure to maintain the stability of its protein structure.

Secondly, the special photosynthetic pathway of *D. officinale* may responsible to the adaptive evolution of *DenHsp70*s. *D. officinale* is a facultative CAM plant and the C3 pathway can be induced by controlling the growing environment (Zhang et al., 2014). Due to the importance of *cis*-elements in gene promoters for plant responses to environmental stresses (Yamaguchi-Shinozaki and Shinozaki, 2005), we further identified them in the putative promoter regions of *DenHsp70*s, and found that light-responsive elements were the most abundant. Since light is an important condition for photosynthesis, we believed that *Hsp70*s plays an important role in *D. officinale* photosynthesis, leading to the adaptive evolution of *DenHsp70*s.

Thirdly, it may partly enhance the heat dissipation capacity of plants. In previous studies, most of eleven chloroplast genome-encoded *ndh* genes (cp-*ndh*), which contribute to plant heat dissipation, were independently lost in *D. officinale* (Lin et al., 2017). However, *Hsp70B* has shown its abilities in the molecular protection of the photosystem reaction centers during photoinhibition and in the process of photosystem repair (Schroda et al., 1999). Moreover, a significant positive relationship between *Hsp70* expression and the acquisition of thermotolerance has been identified (Lee et al., 2009), leading to increased heat and drought stress tolerance in plants (Alvim et al., 2001). Therefore, we speculated that *Hsp70* genes play a more important role in adverse habitat, special photosynthetic pathway and heat dissipation.

## Conclusion

In this study, a total of 37 *Hsp20* genes (*DenHsp20*s), 43 *Hsp70* genes (*DenHsp70*s) and 4 *Hsp90* genes (*DenHsp90*s) were identified and confirmed in *D. officinale* genome. The *DenHsp20*s, *DenHsp70*s and *DenHsp90*s were randomly localized on different chromosomes, and they were classified into 8,4 and 2 subfamilies, respectively, based on the phylogenetic analysis and cellular locations. Moreover, gene structure, molecular evolution, interaction network and expression profiles were comprehensively reported. 13 duplicate gene pairs were identified in *DenHsp70*s, 7 of them were positively selected. These findings provided important information on the evolution of *Hsp70* genes in *D. officinale*. The interaction network and expression profiles were analyzed to provide information on the function in stress response. This work would aid in elucidating the further functional characterizations of *DenHsp20*s, *DenHsp70*s and *DenHsp90*s in the future.

## Data availability statement

The original contributions presented in the study are included in the article/Supplementary material, further inquiries can be directed to the corresponding authors.

## Author contributions

XD, ZN, and JC designed the study. HW, QX, and WL performed the experiments. HW and ZD analyzed the data. MW and YD collected the materials. HW wrote the manuscript. All authors contributed to the article and approved the submitted version.

## Funding

Our work was funded by the National Natural Science Foundation of China (Grant nos. 32070353 and 31900268), Forestry Science and Technology Innovation and Promotion Project of Jiangsu Province [LYKJ(2021)12], Natural Science Foundation of Jiangsu Province (BK20190699), and Foundation of Key Laboratory of National Forestry and Grassland Administration for Orchid Conservation and Utilization (OC202102).

## Conflict of interest

The authors declare that the research was conducted in the absence of any commercial or financial relationships that could be construed as a potential conflict of interest.

## Publisher’s note

All claims expressed in this article are solely those of the authors and do not necessarily represent those of their affiliated organizations, or those of the publisher, the editors and the reviewers. Any product that may be evaluated in this article, or claim that may be made by its manufacturer, is not guaranteed or endorsed by the publisher.

## Footnotes

^1^ https://pfam.xfam.org/

^2^ http://pdslab.biochem.iisc.ernet.in/hspir/

^3^ https://www.ncbi.nlm.nih.gov/Structure/cdd/wrpsb.cgi

^4^ http://smart.embl-heidelberg.de/

^5^ https://meme-suite.org/meme/

^6^ http://gsds.gao-lab.org/index.php

^7^ https://bioinformatics.psb.ugent.be/webtools/plantcare/html/

^8^ https://www.ncbi.nlm.nih.gov/sra

^9^ https://swissmodel.expasy.org/

^10^ http://string-db.org/

## Supplementary material

SUPPLEMENTARY FIGURE 1 Sequence logo of the Hsp20, Hsp70 and Hsp90 proteins motifs. The height of each amino acid represents the relative frequency of the amino acid at that position.

SUPPLEMENTARY FIGURE 2 Multiple sequence alignment of members of the *D. officinale Hsp20*, *Hsp70* and *Hsp90* family. The multiple alignment was generated using DNAMAN_9. The positions of conserved functional domains are named above the align sequences.

SUPPLEMENTARY FIGURE 3 *Cis*-acting elements in the promoter regions of *Hsp20*, *Hsp70* and *Hsp90* genes in *D. officinale* and statistical summary. **(A)** *Cis*-elements with similar functions are displayed in the same color. **(B)** The detailed percentages of each type of *cis*-elements. **(C)** The percentage of three kinds of *cis*-elements.
