# Supplementary figures and images for: Genome-wide identification and expression analysis of the Hsp20, Hsp70 and Hsp90 gene family in Dendrobium officinale
Source: Front Plant Sci. 2022 Aug 10;13:979801. doi: 10.3389/fpls.2022.979801 (PMC9399769; doi:10.3389/fpls.2022.979801)

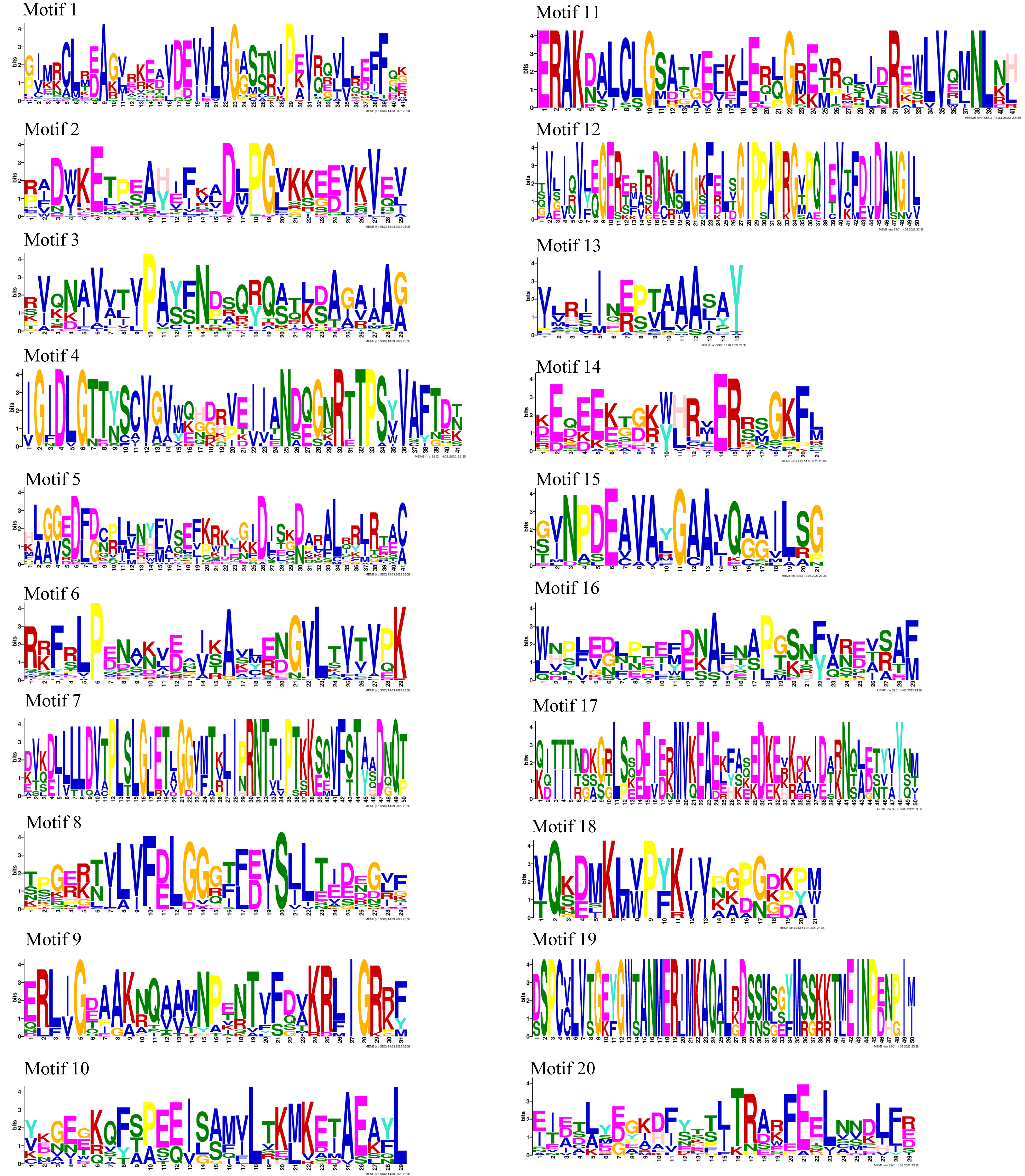

Supplement: SUPPLEMENTARY FIGURE 1 — Sequence logo of the Hsp20, Hsp70 and Hsp90 proteins motifs. The height of each amino acid represents the relative frequency of the amino acid at that position. [file Image_1.TIF]

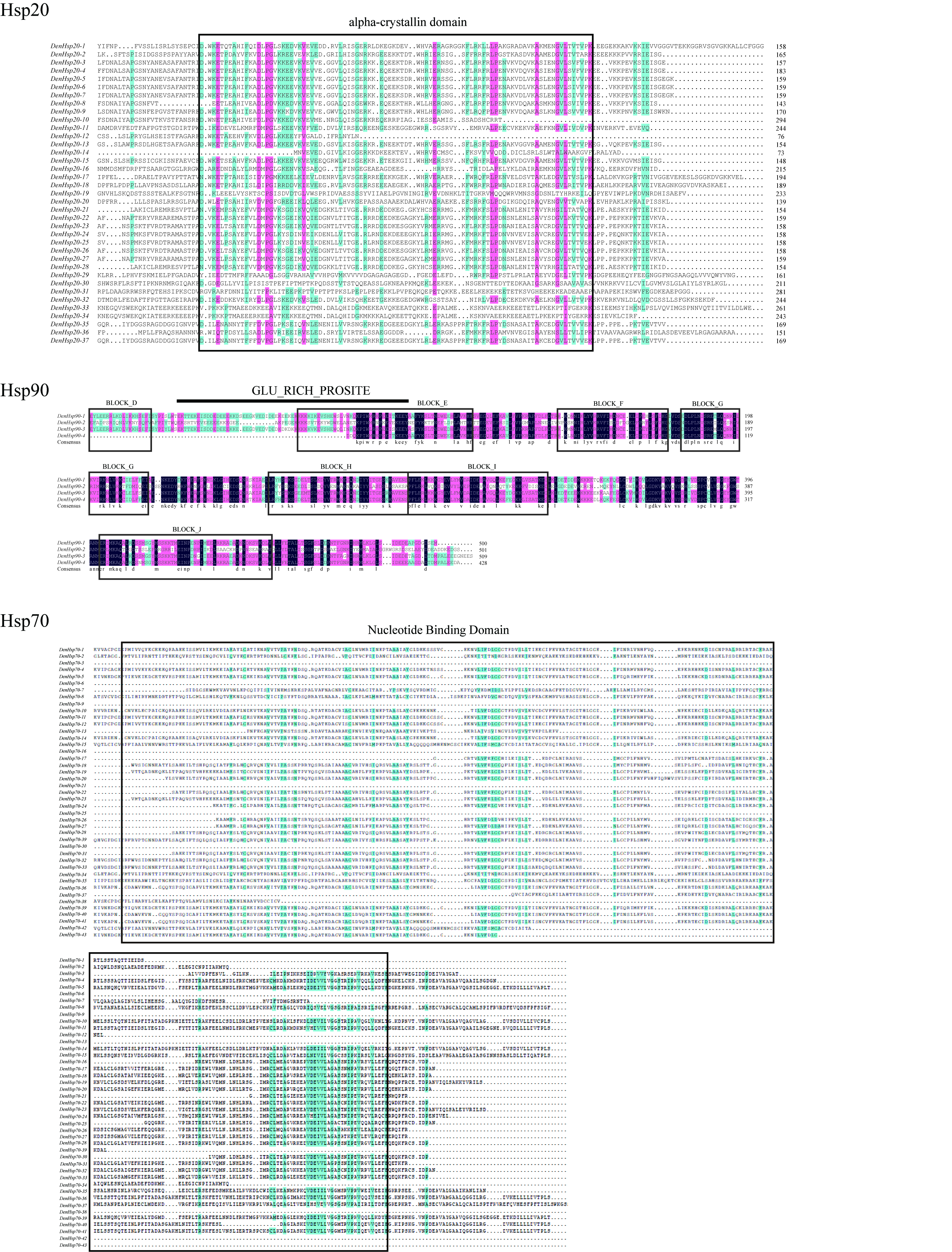

Supplement: SUPPLEMENTARY FIGURE 2 — Multiple sequence alignment of members of the D. officinale Hsp20, Hsp70 and Hsp90 family. The multiple alignment was generated using DNAMAN_9. The positions of conserved functional domains are named above the align sequences. [file Image_2.TIF]

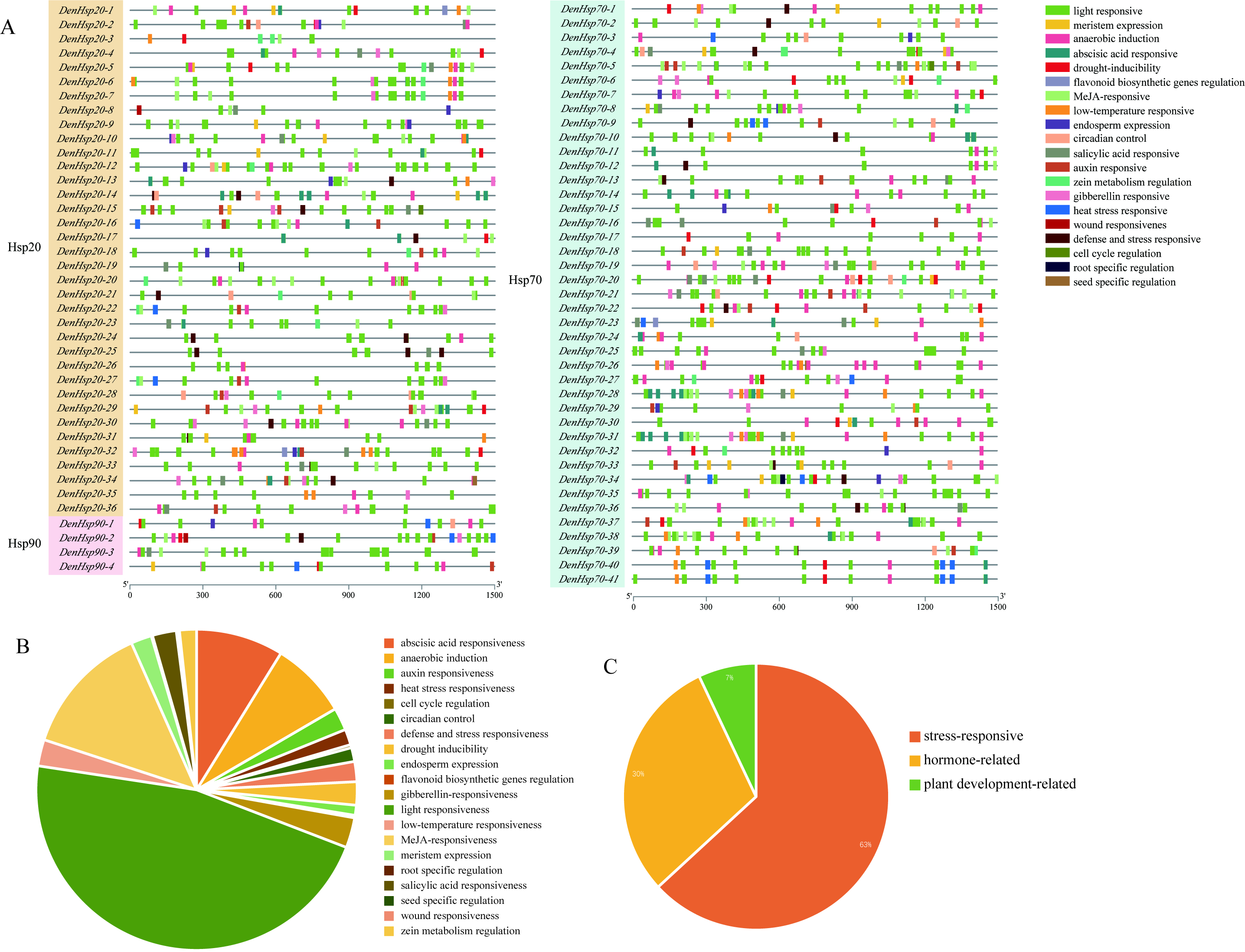

Supplement: SUPPLEMENTARY FIGURE 3 — Cis-acting elements in the promoter regions of Hsp20, Hsp70 and Hsp90 genes in D. officinale and statistical summary. (A) Cis-elements with similar functions are displayed in the same color. (B) The detailed percentages of each type of cis-elements. (C) The percentage of three kinds of cis-elements. [file Image_3.TIF]
