# Supplementary material for: Genome-wide identification and expression analysis of the Hsp20, Hsp70 and Hsp90 gene family in Dendrobium officinale
Source: Front Plant Sci. 2022 Aug 10;13:979801. doi: 10.3389/fpls.2022.979801 (PMC9399769; doi:10.3389/fpls.2022.979801)
Supplement: Supplementary file 4 [file Table_1.DOCX]

| **Table S1** The characteristics of HSP20, HSP70 and HSP90 members identified in *Dendrobium officinale* | | | | | | | |
| --- | --- | --- | --- | --- | --- | --- | --- |
| Gene Family | Gene Name | Gene ID | Protein | Molecular weight（kDa） | Theoretical pI | Aliphatic index | Grand average of hydropathicity（GRAVY） |
| HSP20 | *DenHsp20-1* | *Dof001098* | 159 | 17493.24 | 9.3 | 88.18 | -0.431 |
|  | *DenHsp20-2* | *Dof002521* | 165 | 18865.36 | 8.52 | 66.12 | -0.651 |
|  | *DenHsp20-3* | *Dof003464* | 157 | 17878.14 | 5.84 | 75.67 | -0.655 |
|  | *DenHsp20-4* | *Dof003465* | 183 | 20485.88 | 5.75 | 71.31 | -0.657 |
|  | *DenHsp20-5* | *Dof003469* | 159 | 18040.46 | 6.21 | 81.45 | -0.586 |
|  | *DenHsp20-6* | *Dof003471* | 159 | 18000.43 | 6.21 | 81.45 | -0.551 |
|  | *DenHsp20-7* | *Dof003473* | 159 | 17984.43 | 6.21 | 82.7 | -0.533 |
|  | *DenHsp20-8* | *Dof003474* | 143 | 16196.45 | 5.47 | 84.41 | -0.493 |
|  | *DenHsp20-9* | *Dof003476* | 170 | 19168.98 | 8.71 | 83.59 | -0.546 |
|  | *DenHsp20-10* | *Dof003477* | 294 | 33320.28 | 9.06 | 72.93 | -0.478 |
|  | *DenHsp20-11* | *Dof003771* | 244 | 27693.76 | 9.34 | 71.8 | -0.568 |
|  | *DenHsp20-12* | *Dof006016* | 76 | 8712.91 | 4.92 | 79.61 | -0.228 |
|  | *DenHsp20-13* | *Dof006017* | 154 | 17249.39 | 5.71 | 68.31 | -0.589 |
|  | *DenHsp20-14* | *Dof006026* | 73 | 8422.76 | 5.3 | 96.03 | -0.119 |
|  | *DenHsp20-15* | *Dof006572* | 148 | 16855.2 | 6.6 | 75.61 | -0.484 |
|  | *DenHsp20-16* | *Dof007869* | 215 | 24079.23 | 5.85 | 79.02 | -0.565 |
|  | *DenHsp20-17* | *Dof010207* | 194 | 21973.23 | 5.59 | 95.88 | -0.338 |
|  | *DenHsp20-18* | *Dof010209* | 189 | 20995.14 | 6.86 | 93.97 | -0.275 |
|  | *DenHsp20-19* | *Dof011224* | 233 | 25942 | 5.82 | 77.77 | -0.512 |
|  | *DenHsp20-20* | *Dof011973* | 139 | 15145.42 | 8.09 | 96.19 | -0.235 |
|  | *DenHsp20-21* | *Dof016350* | 154 | 17518.2 | 6.76 | 75.26 | -0.599 |
|  | *DenHsp20-22* | *Dof016351* | 159 | 18039.63 | 5.42 | 77.8 | -0.629 |
|  | *DenHsp20-23* | *Dof016352* | 158 | 17839.43 | 6.4 | 75.82 | -0.542 |
|  | *DenHsp20-24* | *Dof016353* | 158 | 18140.81 | 5.01 | 92.41 | -0.458 |
|  | *DenHsp20-25* | *Dof016354* | 158 | 18140.81 | 5.01 | 92.41 | -0.458 |
|  | *DenHsp20-26* | *Dof016355* | 158 | 17777.36 | 6.4 | 76.46 | -0.541 |
|  | *DenHsp20-27* | *Dof016356* | 159 | 17941.59 | 5.62 | 78.43 | -0.551 |
|  | *DenHsp20-28* | *Dof016358* | 154 | 17518.2 | 6.76 | 75.26 | -0.599 |
|  | *DenHsp20-29* | *Dof019210* | 161 | 17405.64 | 4.77 | 77.45 | -0.094 |
|  | *DenHsp20-30* | *Dof019234* | 211 | 23216.61 | 8.85 | 87.3 | -0.305 |
|  | *DenHsp20-31* | *Dof019235* | 281 | 31429.9 | 5.46 | 54.63 | -1.023 |
|  | *DenHsp20-32* | *Dof020857* | 244 | 27321.13 | 6.66 | 78.69 | -0.488 |
|  | *DenHsp20-33* | *Dof022817* | 261 | 30110.91 | 5.13 | 66.51 | -0.988 |
|  | *DenHsp20-34* | *Dof022820* | 243 | 28261.13 | 5.59 | 67.45 | -0.989 |
|  | *DenHsp20-35* | *Dof023706* | 169 | 18684.1 | 5.15 | 85.33 | -0.484 |
|  | *DenHsp20-36* | *Dof025018* | 151 | 17036.54 | 6.15 | 99.54 | -0.156 |
|  | *DenHsp20-37* | *Dof025693* | 169 | 18684.1 | 5.15 | 85.33 | -0.484 |
| HSP70 | *DenHsp70-1* | *Dof002577* | 284 | 31109.2 | 7.5 | 85.53 | -0.248 |
|  | *DenHsp70-2* | *Dof003997* | 610 | 67598.2 | 4.93 | 85.87 | -0.403 |
|  | *DenHsp70-3* | *Dof003999* | 72 | 7944.1 | 4.79 | 108.19 | -0.076 |
|  | *DenHsp70-4* | *Dof004000* | 383 | 42312.8 | 6.92 | 82.27 | -0.317 |
|  | *DenHsp70-5* | *Dof004107* | 608 | 67569.1 | 4.91 | 87.88 | -0.500 |
|  | *DenHsp70-6* | *Dof005968* | 56 | 6123.7 | 4.14 | 80.00 | -0.309 |
|  | *DenHsp70-7* | *Dof007029* | 203 | 22362.3 | 6.53 | 94.19 | -0.095 |
|  | *DenHsp70-8* | *Dof009462* | 657 | 73484.8 | 6.26 | 83.99 | -0.300 |
|  | *DenHsp70-9* | *Dof012468* | 65 | 7089.8 | 4.4 | 82.46 | -0.092 |
|  | *DenHsp70-10* | *Dof013104* | 595 | 64020.1 | 4.77 | 92.20 | -0.258 |
|  | *DenHsp70-11* | *Dof014409* | 434 | 47727.1 | 6.1 | 85.35 | -0.271 |
|  | *DenHsp70-12* | *Dof014411* | 272 | 29869.8 | 8.2 | 81.36 | -0.325 |
|  | *DenHsp70-13* | *Dof014415* | 83 | 8638.8 | 4.37 | 114.94 | 0.452 |
|  | *DenHsp70-14* | *Dof014952* | 595 | 64110.2 | 4.75 | 93.70 | -0.252 |
|  | *DenHsp70-15* | *Dof015686* | 430 | 46356.3 | 6.85 | 110.05 | 0.198 |
|  | *DenHsp70-16* | *Dof017093* | 65 | 7472.6 | 8.24 | 95.85 | -0.082 |
|  | *DenHsp70-17* | *Dof017097* | 165 | 18349.2 | 7.34 | 100.36 | 0.145 |
|  | *DenHsp70-18* | *Dof017098* | 252 | 28431.2 | 6.4 | 100.24 | -0.092 |
|  | *DenHsp70-19* | *Dof017100* | 266 | 29250.4 | 4.66 | 107.03 | 0.117 |
|  | *DenHsp70-20* | *Dof017101* | 252 | 28413.6 | 8.63 | 109.88 | 0.089 |
|  | *DenHsp70-21* | *Dof017102* | 45 | 5051.8 | 4.51 | 93.11 | 0.016 |
|  | *DenHsp70-22* | *Dof017103* | 239 | 26703.6 | 6.27 | 110.21 | 0.192 |
|  | *DenHsp70-23* | *Dof017105* | 267 | 29624.9 | 4.92 | 108.43 | 0.085 |
|  | *DenHsp70-24* | *Dof017107* | 231 | 25500.5 | 8.3 | 103.46 | 0.136 |
|  | *DenHsp70-25* | *Dof017113* | 78 | 8949.4 | 8.99 | 105.00 | -0.185 |
|  | *DenHsp70-26* | *Dof017114* | 219 | 24094.8 | 7.43 | 111.42 | 0.176 |
|  | *DenHsp70-27* | *Dof017115* | 219 | 24132.7 | 8.34 | 106.94 | 0.092 |
|  | *DenHsp70-28* | *Dof017149* | 241 | 26784.6 | 7.73 | 99.54 | -0.024 |
|  | *DenHsp70-29* | *Dof017151* | 180 | 20162.6 | 8.07 | 85.11 | -0.217 |
|  | *DenHsp70-30* | *Dof017152* | 61 | 6929.9 | 4.99 | 102.13 | -0.152 |
|  | *DenHsp70-31* | *Dof017164* | 236 | 26242 | 8.11 | 100.00 | -0.017 |
|  | *DenHsp70-32* | *Dof017445* | 274 | 30530.8 | 7.86 | 98.21 | 0.031 |
|  | *DenHsp70-33* | *Dof017446* | 266 | 29697.6 | 5.15 | 100.45 | 0.039 |
|  | *DenHsp70-34* | *Dof019012* | 302 | 33551.9 | 4.75 | 85.89 | -0.558 |
|  | *DenHsp70-35* | *Dof019160* | 391 | 43174.1 | 5.38 | 96.29 | -0.163 |
|  | *DenHsp70-36* | *Dof019558* | 431 | 46472.9 | 8.42 | 95.24 | -0.141 |
|  | *DenHsp70-37* | *Dof020203* | 484 | 54334.2 | 4.89 | 82.38 | -0.443 |
|  | *DenHsp70-38* | *Dof020204* | 142 | 15272.5 | 7.29 | 99.58 | 0.139 |
|  | *DenHsp70-39* | *Dof021402* | 608 | 67742.6 | 4.95 | 87.24 | -0.513 |
|  | *DenHsp70-40* | *Dof023904* | 576 | 62165.1 | 4.97 | 93.47 | -0.249 |
|  | *DenHsp70-41* | *Dof023917* | 543 | 58977.5 | 5.16 | 93.06 | -0.278 |
|  | *DenHsp70-42* | *Dof026476* | 151 | 16656.6 | 8.9 | 108.54 | 0.384 |
|  | *DenHsp70-43* | *Dof025759* | 196 | 21476.6 | 8.87 | 94.54 | -0.236 |
| HSP90 | *DenHsp90-1* | *Dof002881* | 500 | 58234.8 | 5.05 | 80.48 | -0.786 |
|  | *DenHsp90-2* | *Dof005585* | 501 | 58069 | 4.65 | 78.98 | -0.700 |
|  | *DenHsp90-3* | *Dof010626* | 510 | 59311 | 4.86 | 79.69 | -0.784 |
|  | *DenHsp90-4* | *Dof017012* | 428 | 49392.1 | 5.04 | 84.95 | -0.591 |
