# Supplementary material for: Genome-wide identification and expression analysis of the Hsp20, Hsp70 and Hsp90 gene family in Dendrobium officinale
Source: Front Plant Sci. 2022 Aug 10;13:979801. doi: 10.3389/fpls.2022.979801 (PMC9399769; doi:10.3389/fpls.2022.979801)
Supplement: Supplementary file 5 [file Table_2.DOCX]

Table S2 Ka, Ks and Ka/Ks values for duplication gene pairs in *Dendrobium officinale*

| Seq_1 | Seq_2 | Ka | Ks | Ka/Ks | Duplication type |
| --- | --- | --- | --- | --- | --- |
| DenHsp20-3 | DenHsp20-4 | 0.0288516 | 0.182468 | 0.158119 | Tandem duplication |
| DenHsp20-3 | DenHsp20-5 | 0.0423036 | 0.629600 | 0.0671912 | Tandem duplication |
| DenHsp20-3 | DenHsp20-6 | 0.0420341 | 0.628976 | 0.0668294 | Tandem duplication |
| DenHsp20-3 | DenHsp20-7 | 0.0485131 | 0.640711 | 0.0757176 | Tandem duplication |
| DenHsp20-3 | DenHsp20-9 | 0.1718140 | 0.762423 | 0.225353 | Tandem duplication |
| DenHsp20-4 | DenHsp20-5 | 0.0685647 | 0.640447 | 0.107058 | Tandem duplication |
| DenHsp20-4 | DenHsp20-6 | 0.0686336 | 0.672358 | 0.102079 | Tandem duplication |
| DenHsp20-4 | DenHsp20-7 | 0.0686761 | 0.696409 | 0.0986146 | Tandem duplication |
| DenHsp20-5 | DenHsp20-6 | 0.0049443 | 0.0728638 | 0.0678567 | Tandem duplication |
| DenHsp20-5 | DenHsp20-7 | 0.0106330 | 0.087752 | 0.121171 | Tandem duplication |
| DenHsp20-6 | DenHsp20-7 | 0.0050522 | 0.0129176 | 0.391112 | Tandem duplication |
| DenHsp20-6 | DenHsp20-9 | 0.1791940 | 0.814981 | 0.219874 | Tandem duplication |
| DenHsp20-7 | DenHsp20-9 | 0.1873980 | 0.838910 | 0.223383 | Tandem duplication |
| DenHsp20-21 | DenHsp20-28 | 9.52E-06 | 0.00951999 | 0.001000 | Tandem duplication |
| DenHsp20-22 | DenHsp20-27 | 0.0171957 | 0.0275679 | 0.623759 | Tandem duplication |
| DenHsp20-22 | DenHsp20-26 | 0.0984053 | 0.732027 | 0.134429 | Tandem duplication |
| DenHsp20-22 | DenHsp20-23 | 0.1049990 | 0.692707 | 0.151578 | Tandem duplication |
| DenHsp20-23 | DenHsp20-26 | 0.0053028 | 0.0508279 | 0.104328 | Tandem duplication |
| DenHsp20-23 | DenHsp20-27 | 0.1064250 | 0.661892 | 0.160789 | Tandem duplication |
| DenHsp20-26 | DenHsp20-27 | 0.0998885 | 0.700082 | 0.142681 | Tandem duplication |
| DenHsp70-1 | DenHsp70-12 | 0.946056 | 1.14994 | 0.822701 | Segmental duplication |
| DenHsp70-5 | DenHsp70-39 | 0.0305446 | 1.66494 | 0.0183457 | Segmental duplication |
| DenHsp70-10 | DenHsp70-14 | 0.0943114 | 2.22029 | 0.0424771 | Segmental duplication |
| DenHsp70-19 | DenHsp70-23 | 0.991257 | 1.02863 | 0.963668 | Tandem duplication |
| DenHsp70-24 | DenHsp70-27 | 0.233487 | 0.244985 | 0.953068 | Tandem duplication |
| DenHsp70-24 | DenHsp70-26 | 0.25634 | 0.233194 | 1.09926 | Tandem duplication |
| DenHsp70-26 | DenHsp70-27 | 0.0434161 | 0.0430908 | 1.00755 | Tandem duplication |
| DenHsp70-28 | DenHsp70-31 | 0.0408689 | 0.0517225 | 0.790157 | Tandem duplication |
| DenHsp70-28 | DenHsp70-32 | 0.201249 | 0.122347 | 1.6449 | Tandem duplication |
| DenHsp70-28 | DenHsp70-33 | 0.209309 | 0.120741 | 1.73354 | Tandem duplication |
| DenHsp70-31 | DenHsp70-32 | 0.1781 | 0.0776771 | 2.29282 | Tandem duplication |
| DenHsp70-31 | DenHsp70-33 | 0.184554 | 0.0753664 | 2.44875 | Tandem duplication |
| DenHsp70-32 | DenHsp70-33 | 0.065504 | 0.02501 | 2.61911 | Tandem duplication |

Synonymous (Ks) and nonsynonymous (Ka) substitution rates of duplicate gene pairs (Ka/Ks ratios)
